# The MAOA Gene Influences the Neural Response to Psychosocial Stress in the Human Brain

**DOI:** 10.3389/fnbeh.2020.00065

**Published:** 2020-05-15

**Authors:** Xiaoqiang Sun, Qingsen Ming, Xue Zhong, Daifeng Dong, Chuting Li, Ge Xiong, Chang Cheng, Wanyi Cao, Jiayue He, Xiang Wang, Jinyao Yi, Shuqiao Yao

**Affiliations:** ^1^Medical Psychological Center, The Second Xiangya Hospital, Central South University, Changsha, China; ^2^Medical Psychological Institute of Central South University, Changsha, China; ^3^National Clinical Research Center for Mental Disorders, Changsha, China; ^4^Department of Psychiatry, The First Affiliated Hospital of Sochoow University, Suzhou, China

**Keywords:** stress, functional magnetic resonance imaging, monoamine oxidase A, cortisol, hippocampus

## Abstract

The stress response is regulated by many mechanisms. Monoamine oxidase A (MAOA) has been related to many mental illnesses. However, few studies have explored the relationship between MAOA and acute laboratory-induced psychosocial stress with functional magnetic resonance imaging (fMRI). In the current study, the Montreal Imaging Stress Task (MIST) and fMRI were used to investigate how MAOA influences the stress response. Increased cortisol concentrations were observed after the task; functional connectivity between the bilateral anterior hippocampus and other brain regions was reduced during stress. MAOA-H allele carriers showed greater deactivation of the right anterior hippocampus and greater cortisol response after stress than did MAOH-L allele carriers. Hippocampal deactivation may lead to disinhibition of the hypothalamic-pituitary-adrenal (HPA) axis and the initiation of stress hormone release under stress. Our results suggest that the MAOA gene regulates the stress response by influencing the right anterior hippocampus.

## Introduction

Stress is a process of adaptation and reaction occurring when a person faces pressure or expected environmental change that threatens to disrupt organismal homeostasis (Ulrich-Lai and Herman, 2009; Lucassen et al., 2014). Stress activates the sympathetic nervous system and the hypothalamic-pituitary-adrenal (HPA) axis, which then maintains or restores homeostasis (Ulrich-Lai and Herman, 2009). The HPA axis is a complex system that may interact with neurotransmitters (Cullinan et al., 2008; Kalbitzer et al., 2013) and the immune system (Leonard, 2005); it is also regulated by the central nervous system, including the prefrontal cortex (PFC), hippocampus, and amygdala (Dedovic et al., 2009). Stress is related closely to health; people who experienced high stress levels in early life are at greater risk of developing mental disorders (Green et al., 2010), and recent stress contributes substantially to subsequent mental illness (Stroud et al., 2008).

Monoamine oxidase A (MAOA) is a mitochondrial enzyme involved in the catabolism of catecholamine neurotransmitters, including norepinephrine, serotonin, and dopamine (Buckholtz and Meyer-Lindenberg, 2008). The MAOA gene, located on the X chromosome (Xp11.23), has emerged as an important genetic factor in relation to mental illness (Fan et al., 2010; Naoi et al., 2018). MAOA transcriptional efficiency is impacted by the variable number of tandem repeats (VNTR) domain of the gene, such that lower expression of MAOA is seen in carriers of 2, 3, and 5 repeats (L alleles; MAOA-L) and higher expression of MAOA is seen in carriers of 3.5 and 4 repeats (H alleles; MAOA-H; Sabol et al., 1998). At present, the most consistent view is that MAOA-L carriage is associated with aggression, impulsive behavior, and antisocial personality disorders, especially in individuals who experienced childhood abuse (Caspi et al., 2002; Buckholtz and Meyer-Lindenberg, 2008; Byrd and Manuck, 2014). Although some evidence indicates that MAOA-H carriers are at greater risk of depression (Beach et al., 2010; Fan et al., 2010; Naoi et al., 2018), some researchers have expressed contrary opinions (Brummett et al., 2007; Marmorstein and Hart, 2011); besides, gene × sex interaction appears to affect the development of depression (Priess-Groben and Hyde, 2013) and anxiety (Voltas et al., 2015). Stress and its interaction with MAOA play important roles in the occurrence of these diseases.

Psychosocial stress, induced by social threats, is often caused by factors such as companion or family relationships, or occupational or financial pressure. It can put pressure on an individual, with psychosocial demands (Booth et al., 2015). Jabbi et al. (2007) indicated that MAOA influences the regulation of the HPA axis response to acute psychological stress and endocrine challenges; they found that male MAOA-L carriers showed higher subjective stress levels than did their MAOA-H-carrying counterparts in a psychological stress task, whereas females showed the opposite pattern. In a combined dexamethasone and corticotrophin-releasing hormone challenge, MAOA-L carriers showed lesser glucocorticoid responses than did MAOA-H carriers, and males had greater responses than did females (Jabbi et al., 2007). Brummett et al. (2008) found that MAOA-L individuals under chronic stress showed blunted HPA axis responses during the day, reflecting risk of HPA exhaustion. Existing evidence for the effect of MAOA on the HPA axis under stress conditions is inconsistent, possibly due to the use of different stimulation types (acute stress, chronic stress, and endocrine challenge) in previous studies.

Glucocorticoids are involved in the regulation of brain MAOA (Ou et al., 2006), through pathways such as those involving Kruppel-like factor 11 (KLF11) and cell division cycle-associated 7-like protein (R1). Glucocorticoids can increase KLF11 messenger RNA (mRNA) and protein levels, and KLF11 overexpression increases MAOA gene expression and enzymatic activity (Grunewald et al., 2012). R1 interacts with Sp1-binding sites in the MAOA promoter and represses transcription of the gene (Chen et al., 2005); glucocorticoids enhance MAOA expression by regulating R1 translocation and interacting with Sp1 or the R1 transcription factor on Sp1-binding sites of the MAOA promoter (Ou et al., 2006). In the brain, 5-hydroxytryptamine (5-HT) is degraded primarily by MAOA (Higuchi et al., 2017). Corticosterone decreases 5-HT_1A_ receptor expression in the hippocampus and increases 5-HT_2_ receptor expression in the frontal cortex (Takao et al., 1997). Maternal separation increases hippocampal serotonin turnover and glucocorticoid receptor expression (Higuchi et al., 2017). Animals under chronic stress showed increased MAOA levels and 5-hydroxyindoleacetic acid/5-HT ratios, which indicate serotonin turnover (Winberg and Lepage, 1998; Higuchi et al., 2019); long-term socially defeated mice showed elevated MAOA mRNA and serotonin transporter levels (Filipenko et al., 2002). 5-HT signals also influence the HPA axis; 5-HT inhibits hippocampal mineralocorticoid receptor synthesis (Semont et al., 1999), and its intracerebroventricular injection induces an increase in corticotropin-releasing hormone gene expression in the hypothalamic paraventricular nucleus in rats (Kageyama et al., 1998). However, acute social stress significantly reduces brain MAOA binding in humans (Soliman et al., 2012), suggesting that chronic and acute stress have different effects on brain MAOA levels. Taken together, these findings suggest that chronic stress and glucocorticoids are related to elevated MAOA levels and interact with the 5-HT system to regulate stress responses; acute stress has been found to reduce MAOA binding, activity, and protein levels in the brain, which may be an adaptive process and promote coping behavior in response to mild acute stress (Soliman et al., 2012).

The influence of the MAOA gene on the HPA axis may occur through the hippocampus and other limbic areas. Corticosteroids influence cells in the hippocampus through hippocampal glucocorticoid receptors (Bogdan et al., 2016), and chronic stress can cause hippocampal atrophy (Higuchi et al., 2017). Hippocampal MAOA levels and MAOA gene transcription were found to be significantly higher in burned mice than in control mice, and a MAOA inhibitor reversed depressive-like behavior and decreased the elevated expression of MAOA in the hippocampus in the burned mice (Wang et al., 2017a). Increased MAOA protein levels in the hippocampus and depressive-like symptoms were also found in sleep-deprived mice (Wang et al., 2017b). The inhibition of MAOA density by MAOA inhibitors has been observed in many brain regions, including the hippocampus, anterior cingulate cortex (ACC), PFC, and midbrain (Sacher et al., 2011). Selective MAOA inhibition reversed reduced hippocampal adult neurogenesis and dendritic plasticity induced by chronic stress in rats (Morais et al., 2014; Stefanovic et al., 2016). In humans, childhood stress interacts with the MAOA gene, resulting in differences in hippocampal activation during emotional tasks (Holz et al., 2016). Taken together, these findings indicate that the hippocampal concentration of MAOA is affected by various factors and may affect glucocorticoid receptors by targeting serotonin, and that the MAOA gene may influence the hippocampal MAOA concentration and subsequent effects on stress through the hippocampus.

Functional magnetic resonance imaging (fMRI) studies have shown that MAOA VNTR may impact brain functioning during various cognitive tasks. MAOA-L carriers showed greater responses in the amygdala, hippocampus, and dorsal ACC (dACC), and less activity in the PFC, during emotional arousal than did MAOA-H carriers (Meyer-Lindenberg et al., 2006; Denson et al., 2014; Holz et al., 2016); they have also shown greater orbitofrontal cortex activation during working memory task performance (Cerasa et al., 2008), and deficient activation of the ACC/dACC during an inhibitory control task (Meyer-Lindenberg et al., 2006; Passamonti et al., 2006; Sun et al., 2018). However, to our knowledge, no study has investigated whether the MAOA genotype influences brain activity during stress tasks.

In the current study, we administered an acute laboratory psychosocial stress task to healthy adults during fMRI. The Montreal Imaging Stress Task (MIST) used in this study is thought to be valid for the induction of stress, and has been used widely (Dedovic et al., 2005). We collected saliva for the measurement of cortisol levels before, during, and after the stress task. We hypothesized that significant differences would exist between MAOA genotype groups in areas of the brain associated with stress, including limbic areas and the ventromedial PFC (vmPFC), and that stress would influence the functional connectivity between the anterior hippocampus and other brain areas.

## Materials and Methods

Study participants were recruited from two colleges and the community in Changsha, Hunan, China, using posters and advertisements. All subjects were Chinese of Han ethnicity. The subjects underwent psychiatric evaluation using the Structured Clinical Interview for DSM-IV-TR Axis I Disorders, Patient Edition, administered by two independent psychiatrists. Exclusion criteria were as follows: (1) current or history of a DSM-IV-TR axis I disorder; (2) history of alcohol/substance abuse; (3) history of severe head injury or neurological illness, or a neurosurgical procedure; (4) IQ <80, assessed by the Chinese version of the Wechsler Adult Intelligence Scale; and (5) female sex. Each participant was told the purpose of the study and signed an informed consent form. The study complied with the Declaration of Helsinki and was approved by the Ethics Committee of the Second Xiangya Hospital of Central South University.

### Genotype

Trained researchers collected DNA samples from participants’ exfoliated buccal cells with TIANamp Swab DNA kits (TIANGEN Biotech, Beijing, China), following standard procedures. MAOA-upstream VNTR genotypes were analyzed using polymerase chain reaction (PCR) in 25-μl reaction volumes containing 1 μl of DNA, 12.5 μl of GoTaq Green Master Mix (Promega Company, Madison, WI, USA), 1.2 μM of each primer (primer sequences, 5′-ACAGCCTGACCGTGGAGAAG-3′ and 5′-GAACGGACGCTCCATTCGGA-3′), and 9.5 μl of double-distilled H_2_O. The amplification protocol was as follows: warming to 95°C for 2 min, followed by 40 cycles at 94°C for 30 s, 59°C for 90 s, and 72°C for 60 s (Zhang et al., 2016). The reactions were performed in a Gene Amp PCR system 2400 (Applied Biosystems, Waltham, MA, USA). The PCR products were separated using electrophoreses on a 3% agarose gel, stained with Du Red (Biosharp, Carlsbad, CA, USA), and visualized under ultraviolet transillumination. Product sizes were determined by comparison with molecular length standards (50 bp ladder; TIANGEN Biotech).

MAOA alleles with 2, 3, and 5 repeats were categorized as having low activity (MAOA-L), whereas those with 3.5 and 4 repeats were categorized as having high activity (MAOA-H; Ma et al., 2018). On this basis, the subjects were divided into MAOA-L and MAOA-H groups. Two individuals independently performed allele classification; a third investigator reviewed the classifications when a consensus was lacking, and samples were rerun when the results were ambiguous.

### Stress Task

**Figure 1:**
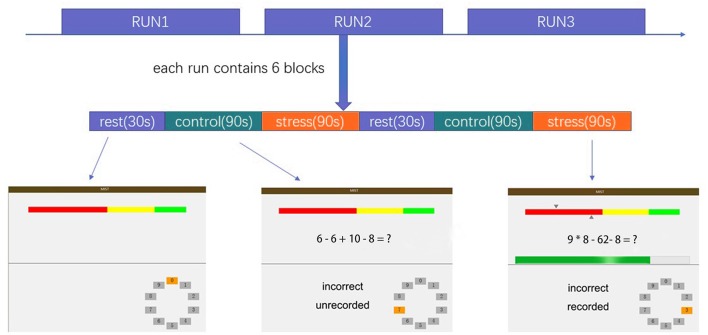
Overview of the stress task. The colored bar at the top of the display represents the number of the subject’s correct responses (top arrow = average performance, bottom arrow = individual subject’s performance).

The MIST was administered using a block design with three imaging runs. Each run lasted 7 min and consisted of six blocks with three different conditions: a rest condition (30 s), during which subjects were asked only to watch the screen; a control condition (90 s), in which subjects were asked to solve arithmetic problems with no time requirement; and a stressful experimental condition (90 s), in which subjects were asked to solve arithmetic problems with a time limit while a countdown bar was displayed on the screen. The order of presentation in each run is rest–control–experimental–rest–control–experimental, with no interval between blocks. The experimental program was projected onto the screen in front of the subject, who responded by pressing buttons (two buttons for answer selection and one button to confirm the selection). The overall experimental procedure is shown in Figure 1 and described in the Supplementary Material. Investigators provided scripted negative verbal feedback after each run segment *via* headphones.

### Psychological and Physiological Measures

All subjects completed the State-Trait Anxiety Inventory, a widely used scale in psychological research (Li et al., 2019), before the stress task. Participants’ subjective stress levels were assessed before the task began and after they had completed the three runs using a 0–10 visual analog scale (0 = absence of stress, 10 = maximal stress) with oral reporting. Changes in subjective stress were determined by subtracting the pre-stress from the post-stress values (Ming et al., 2017).

Salivary cortisol, a frequently used biomarker of psychological stress that correlates significantly with serum and plasma cortisol levels, was measured (Kirschbaum and Hellhammer, 1989; Aardal and Holm, 1995; Jung et al., 2014). Salivary cortisol measurement is used widely with psychological stress tasks such as the MIST and the Trier Social Stress Test (Dedovic et al., 2005; Vors et al., 2018). Saliva samples were collected with a Salivette (Sarstedt, Nümbrecht, Germany) at participants’ time of arrival (Cort1), after 30 min of rest (Cort2), just before entering the fMRI scanner (Cort3), during anatomical imaging (Cort4), after MIST runs 1–3 (Cort5–7), and upon leaving the scanner (Cort8). To control for circadian fluctuations, scanning was performed between 2:00 and 5:00 pm. Cortisol concentrations were detected with a human cortisol enzyme-linked immunosorbent assay kit (Bio-Swamp, Shanghai, China). We subtracted Cort4 (the baseline cortisol level before the stress task) from Cort8 (the highest cortisol level after the stress task) and used this value to represent the summary measure of cortisol responses, as this method did not rely on the exact timing of repeated cortisol measurements (Ming et al., 2017).

### Imaging

Scanning was conducted in a 3.0-T Siemens Magnetom Skyra scanner (Siemens Healthineers, Erlangen, Germany). Blood oxygen level–dependent data were acquired with an echoplanar imaging sequence using the following scanning parameters: repetition time = 2 s, echo time = 30 ms, flip angle = 80°, field of view = 256 × 256 mm^2^, matrix = 64 × 64, voxel size = 4 × 4 × 4 mm^3^, 32 slices, slice thickness = 4 mm, and slice gap = 1 mm.

### Data Preprocessing and Statistical Analysis

The fMRI data were preprocessed in SPM12^1^. First, images were corrected to the middle layer using slice timing and then realigned to the first image from each session with six-parameter rigid-body transformation to correct for movement artifacts. Second, we normalized the images to a standard EPI template (Montreal Neurological Institute coordinate system) with resampling into 3 × 3 × 3-mm^3^ voxels. Finally, the data were smoothed with a Gaussian kernel that was 6 mm full width at half maximum.

The preprocessed data were entered into a general linear model (GLM) in SPM12^1^. The GLM included the three conditions (rest, control, and experimental) and was run using the SPM12 default settings and the canonical hemodynamic response function with a high-pass filter of 210 (total time of a cycle of three conditions). Six motion parameters were included to model movement-correlated effects. In the first-level (within-subject) analyses, we determined the contrast “experimental vs. control” for region activation or deactivation when the subject experienced stress (Ming et al., 2017). The resulting contrast images were used in the second-level (between-subject) analyses. One-sample *t*-tests were used to illustrate brain activation during stress; two-sample *t*-tests were conducted to identify brain areas with different activity levels between the groups. Imaging differences between groups were corrected using the familywise error (FWE) rate at the cluster level with initial uncorrected *p* = 0.001 for multiple comparisons (FWE significance *p* ≤ 0.05). We used the Marsbar toolbox^2^ to extract activation values for brain areas that showed significant differences in two-sample *t*-tests. The demographic characteristics of the subjects and the cortisol concentrations and subjective stress values were analyzed using SPSS 18.0^3^. Correlations of brain region activation with cortisol responses, subjective stress levels, and scale scores were examined using SPSS 18.0. *P*-values < 0.05 were considered to be significant.

A psychophysiological interaction (PPI) analysis was performed to identify brain regions with different levels of anterior hippocampal functional connectivity between stress and control periods (Luo et al., 2016). Our reasons for choosing the bilateral anterior hippocampus were twofold: the hippocampus has an important function during stress regulation, and the MAOA concentration in the hippocampus is associated with stress. The peak voxel coordinates from the one-sample *t*-test results were used as landmarks for individual seed voxels (left: −24 −9 −24, right: 27 −12 −21). A spherical region of interest (ROI) with a 5-mm radius was defined around the peak voxel. The time series of each ROI was extracted and the PPI regressor was calculated as the element-by-element product of the mean-corrected activity of this ROI and a vector coding for the differential effect between the stress and control conditions. The GLM regressor of PPI analysis included the bold signal of ROI, the psychological variable (convolved task conditions), calculated PPI interaction terms, and the six motion parameters. The PPI regressors reflect the interaction between the psychological variable (stress or no stress) and the time course of anterior hippocampus activation. Images reflecting the effects of PPI between the anterior hippocampus and other brain areas reveal brain regions with altered strengths of correlation with the anterior hippocampus during the stress compared with the control condition. The PPI results were corrected using the FWE rate for multiple comparisons (significance at *p* ≤ 0.05).

## Results

From an initial total of 109 subjects, eight were excluded from further analyses due to excessive head motion (≥2 mm or ≥2°). Thus, the final analysis included data from 101 subjects. The genotype distribution in our male sample was consistent with previous reports for Han Chinese samples. The characteristics of the genotype groups are presented in Table 1. The two groups did not differ in age, years of education, state anxiety score, or trait anxiety score.

**Table 1:** Demographic characteristics of study participants.

	L allele carriers (*N* = 58)		H allele carriers (*N* = 43)			
	Mean	SD	Mean	SD	*t*	*p*
Age	21.38	1.56	21.65	3.45	0.531	0.597
Education	14.69	2.05	14.58	1.28	0.306	0.761
State anxiety score	35.98	6.31	35.63	6.37	0.278	0.781
Trait anxiety score	36.55	5.74	35.69	7.31	0.658	0.512

**Figure 2:**
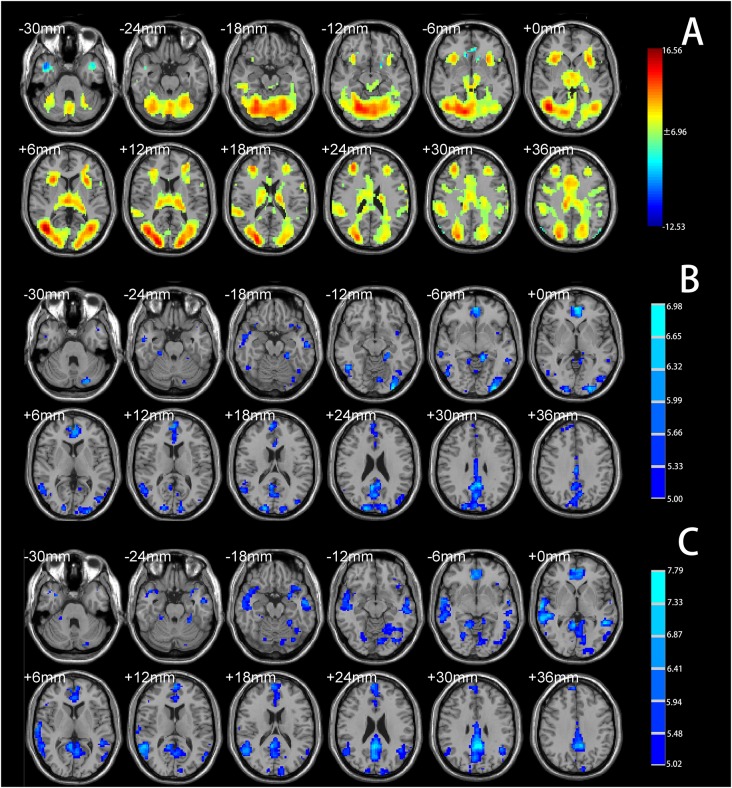
**(A)** Brain regions showing significant activation under the Montreal Imaging Stress Task (MIST) stress condition compared with the control condition (total cohort). **(B)** Brain regions showing significantly decreased functional connectivity with the left anterior hippocampus under the stress condition compared with the control condition (total cohort). **(C)** Brain regions showing significantly decreased functional connectivity with the right anterior hippocampus under the stress condition compared with the control condition (total cohort). Significance was determined based on voxel-wise familywise error (FWE)—corrected *p* < 0.05; the colored bar indicates the *t* statistic.

One-sample *t*-tests identified significant psychosocial stress–induced brain activation in all participants. Psychosocial stress induced by the MIST resulted in extensive activation in the temporal lobe, occipital lobe, frontal lobe, dACC, and insular cortex (Figure 2A, Supplementary Material). Deactivation was detected in the vmPFC, subgenual ACC, anterior hippocampus, temporal pole, and angular gyrus (Figure 2A, Supplementary Material).

PPI analysis showed that both groups had decreased functional connectivity under the stress condition compared with the control condition in the temporal lobe, occipital lobe, limbic lobe, vmPFC, and ACC (Figures 2B,C, Supplementary Material). No brain area showed increased functional connectivity under the stress condition relative to the control condition, indicating that psychosocial stress tended to reduce the functional connectivity of the anterior hippocampus with other brain regions.

**Figure 3:**
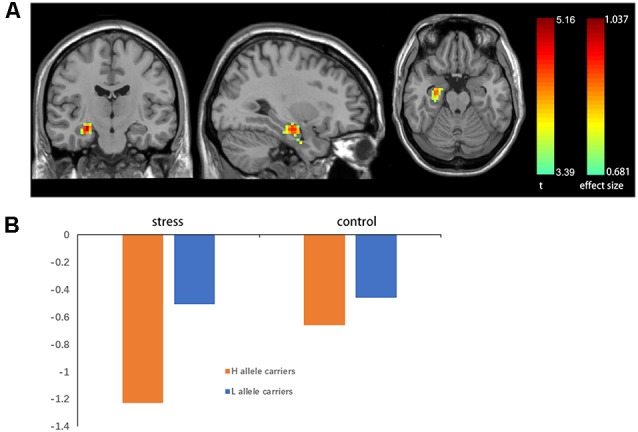
**(A)** Brain activation in MAOA-L and MAOA-H carriers. Significance was determined based on uncorrected *p* < 0.001 (FWE significance at *p* ≤ 0.05). **(B)** Mean beta values for significant clusters (in the right anterior hippocampus) between groups obtained by contrasting the rest condition with the control and stressful conditions.

**Figure 4:**
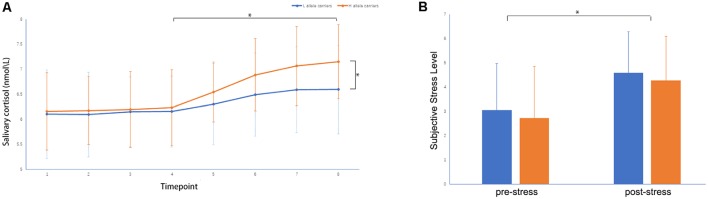
**(A)** Salivary cortisol concentrations and **(B)** subjective stress levels throughout the experiment: (1) on participant arrival, (2) after 30 min rest, (3) upon entering the scanner, (4) during anatomical imaging, (5–7) after MIST runs 1–3, and (8) upon leaving the scanner, *p < 0.05.

**Figure 5:**
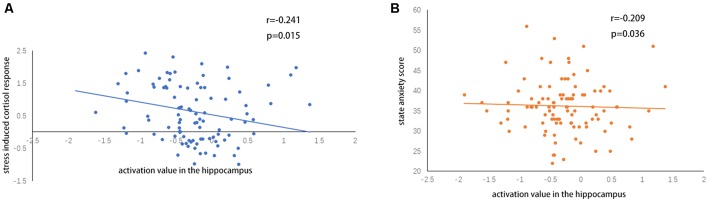
**(A)** Correlations of hippocampal activation under stress with **(A)** stress-induced cortisol responses and **(B)** state anxiety scores.

A two-sample *t*-test showed that MAOA-L carriers had reduced deactivation in the right anterior hippocampus compared with MAOA-H carriers (Table 2, Figure 3). However, no area showed different functional connectivity between groups. The repeated-measures GLM of cortisol concentrations revealed a main effect of time (*F* = 31.592, *p* < 0.001, partial *η*^2^ = 0.242), with the concentration elevated after the stress task (Figure 4A). A main effect of group (*F* = 4.222, *p* = 0.043, partial *η*^2^ = 0.041) and group × time interaction (*F* = 2.175, *p* = 0.016, partial *η*^2^ = 0.032) were also evident, indicating a small, but significant, effect of the genotype (Figure 4A). The repeated-measures GLM of the subjective stress level revealed a main effect of stress (*F* = 60.724, *p* < 0.001, partial *η*^2^ = 0.380; Figure 4B), but no group × stress interaction or main effect of group. The stress level and cortisol results indicate that the MIST successfully induced stress. Cortisol responses and state anxiety scores correlated negatively with activation in the hippocampus in all subjects (*r* = –0.241, *p* = 0.015 and *r* = –0.209, *p* = 0.036, respectively; Figure 5).

**Table 2:** Characteristics of greater activation of the right anterior hippocampus in L allele carriers.

Brain region	Side	MNI coordinates			Cluster size	*t*	*P*_uncorrected_	*P*_FWE corrected_
		*x*	*y*	*z*				
Anterior hippocampus	R	27	−12	−18	135	5.16	<0.001	0.034

## Discussion

In the current study, the cortisol response was elevated in MAOA-H carriers compared with MAOA-L carriers, and MAOA-L carriers showed enhanced neural activity in the right anterior hippocampus compared with MAOA-H carriers. This activity correlated negatively with cortisol responses and state anxiety. PPI analysis revealed decreased functional connectivity between the bilateral anterior hippocampus and brain regions including the limbic lobe, vmPFC, subgenual ACC, and posterior cingulate during stress.

We found decreased activation under stress of limbic system components, including the subgenual ACC, anterior hippocampus, and vmPFC, which are implicated in the regulation of the HPA axis (Ulrich-Lai and Herman, 2009), consistent with findings from other psychosocial stress studies (Pruessner et al., 2008; Kogler et al., 2015). These regions receive and integrate higher-order sensory processing and memory information from subcortical and cortical areas, and provide this information to downstream regions (Ulrich-Lai and Herman, 2009). We also found decreased functional connectivity between the bilateral anterior hippocampus and the vmPFC, subgenual ACC, posterior cingulate, and dACC under the stress condition compared with the control condition. The hippocampus is linked to inhibition of the HPA axis, possibly through inhibition of the paraventricular nucleus of the hypothalamus (Dedovic et al., 2009; Ulrich-Lai and Herman, 2009). Hippocampal stimulation reduces glucocorticoid secretion and hippocampal damage increases stress-induced glucocorticoid secretion in rats, and chronic stress causes atrophy of the hippocampus in humans (McEwen et al., 2016). Under stress, the activity and functional connectivity of the hippocampus may be reduced, disinhibiting the HPA axis and initiating stress hormone release (Pruessner et al., 2008). We found a negative correlation between hippocampal activation and cortisol secretion, which may comprise evidence supporting this possibility. Thus, greater hippocampal deactivation appears to be related to greater cortisol secretion and, in turn, a greater stress response.

Elevated cortisol responses and subjective stress in all participants after the stress task confirmed that the MIST effectively induced stress. The elevated cortisol response in MAOA-H carriers may reflect a greater stress response. In a previous study, male MAOA-L carriers had a greater subjective experience of stress while performing mental arithmetic, which is inconsistent with our result (Jabbi et al., 2007). However, MAOA-H carriers had higher plasma cortisol levels during an endocrine challenge in that study, which may indicate that they were more sensitive to stress (Jabbi et al., 2007). A possible explanation for this inconsistency is that the previous study included male and female participants, and healthy adults and those with depression under medication; this sample diversity, as well as the use of different methods to induce cortisol responses, may be related to the differences in results. We found no group difference in subjective stress, but we did observe differences in a more objective physiological indicator (i.e., cortisol), which may reflect healthy adults’ good regulation and/or self-evaluation of stress.

Our finding of enhanced neural activity in the right anterior hippocampus and reduced cortisol response in MAOA-L carriers compared with MAOA-H carriers may be linked to the influence of the MAOA genotype on the HPA axis. Greater deactivation of the right hippocampus, leading to more disinhibition of the HPA axis and greater stress hormone release, may occur in MAOA-H carriers. Increased MAOA levels in the hippocampus may lead to depressive behavior in mice and rats after stress stimulation (Morais et al., 2014; Wang et al., 2017a). The use of a MAOA inhibitor decreased MAOA levels and restored hippocampal neurogenesis and dendritic plasticity, which are impaired by chronic stress, in rats (Morais et al., 2014). These results suggest that stress leads to higher concentrations of MAOA and subsequent depressive symptoms. However, few studies have examined whether elevated concentrations of MAOA in the hippocampus predispose individuals to acute stress. In a positron emission tomography study, acute social stress induced significant reductions in MAOA binding in the brain, including in the PFC, hippocampus, ACC, thalamus, and midbrain, indicating rapid adaptation (Soliman et al., 2012); stress-induced decreases in MAOA binding and/or activity may contribute to increased monoamine expression, which is associated with normal behavioral stress responses and HPA axis activation (Soliman et al., 2012). MAOA-H carriage may be associated with greater transcriptional efficiency (Sabol et al., 1998), and thus a higher concentration of MAOA in the brain; higher MAOA levels in the hippocampus may lead to less adaptation to acute stress. Our finding of greater cortisol response in MAOA-H carriers may provide evidence supporting this argument. However, causal relationships need to be demonstrated in further experimental studies.

We found only a small effect of group on the cortisol response and no difference between groups in the subjective stress level. A plausible interpretation of these findings is that in our sample of healthy people with normal functional regulation of stress, and given the complexity of the stress system, the influence of the MAOA genotype may interact with other mechanisms, especially those of the COMT and 5HTTLPR genes, which influence the stress tasks (Jabbi et al., 2007; Doornbos et al., 2009; Bouma et al., 2012; Sun et al., 2020), also have shown gene × gene interaction in their effects on HPA axis function during acute stress, thereby generating smaller effects on behavioral phenotypes.

This study has some limitations. First, we did not include environmental factors in the analysis. Monoamine-sensitive developmental periods are related to the occurrence of depression, aggression, and other mental illnesses (Suri et al., 2015). Adverse circumstances in the early developmental years may have an important influence on the stress response in adulthood. Additional research is needed to assess the effects of gene–environment interactions on neural mechanisms, as well as intergene and multigene interactions with environmental factors. Second, as this study included only males, the results may not be applicable to females. The influence of the MAOA genotype on stress appears to differ between males and females (Jabbi et al., 2007). Another limitation is that our sample consisted of healthy people; further investigation is required to determine whether the results can be generalized to broader populations. Lastly, we did not balance the button pressing times of the two experimental conditions; the subjects had sufficient time to solve the math problem and then press the button under the control condition, whereas they did not always solve the problem and press the button in time under the experimental condition. However, we balanced the difficulty of the math problems to keep the accuracy rates under the two conditions within the preset ranges, as in earlier studies (Dedovic et al., 2005), which successfully induced stress.

In conclusion, we found that relative to MAOA-L carriers, MAOA-H carriers had greater cortisol responses and deactivation of the right hippocampus when performing the MIST. This increased right hippocampal deactivation may lead to greater disinhibition of the HPA axis and the initiation of greater stress hormone release under stress in MAOA-H carriers. Future studies should examine gene–environment interactions to better elucidate the importance of the MAOA genotype in stress response and management.

## Data Availability Statement

The raw data supporting the conclusions of this article will be made available by the authors, without undue reservation, to any qualified researcher.

## Ethics Statement

The studies involving human participants were reviewed and approved by Ethics Committee of the Second Xiangya Hospital of Central South University. The patients/participants provided their written informed consent to participate in this study.

## Author Contributions

SY, XW, and JY developed the study. GX, CC, and JH collected the data. CL, XZ, and WC supervised the genotype assessment. XS, QM, and DD analyzed the data. XS drafted the manuscript, and all authors revised the final version of the manuscript and approved it for submission.

## Conflict of Interest

The authors declare that the research was conducted in the absence of any commercial or financial relationships that could be construed as a potential conflict of interest.

## Footnotes

^1^http://www.fil.ion.ucl.ac.uk/spm

^2^http://marsbar.sourceforge.net/

^3^https://www.ibm.com/analytics/us/en/technology/spss/

## References

[B1] Aardal E. Holm A. C. (1995). Cortisol in saliva—reference ranges and relation to cortisol in serum. Eur. J. Clin. Chem. Clin. Biochem. 33, 927–932. 10.1515/cclm.1995.33.12.927 8845424

[B2] Beach S. R. Brody G. H. Gunter T. D. Packer H. Wernett P. Philibert R. A. (2010). Child maltreatment moderates the association of MAOA with symptoms of depression and antisocial personality disorder. J. Fam. Psychol. 24, 12–20. 10.1037/a0018074 20175604 PMC2839928

[B3] Bogdan R. Pagliaccio D. Baranger D. A. Hariri A. R. (2016). Genetic moderation of stress effects on corticolimbic circuitry. Neuropsychopharmacology 41, 275–296. 10.1038/npp.2015.216 26189450 PMC4677127

[B4] Booth J. Connelly L. Lawrence M. Chalmers C. Joice S. Becker C.. (2015). Evidence of perceived psychosocial stress as a risk factor for stroke in adults: a meta-analysis. BMC Neurol. 15:233. 10.1186/s12883-015-0456-4 26563170 PMC4643520

[B5] Bouma E. M. C. Riese H. Doornbos B. Ormel J. Oldehinkel A. J. (2012). Genetically based reduced MAOA and COMT functioning is associated with the cortisol stress response: a replication study. Mol. Psychiatry 17, 119–121. 10.1038/mp.2011.115 21912392

[B6] Brummett B. H. Boyle S. H. Siegler I. C. Kuhn C. M. Surwit R. S. Garrett M. E.. (2008). HPA axis function in male caregivers: effect of the monoamine oxidase-A gene promoter (MAOA-uVNTR). Biol. Psychol. 79, 250–255. 10.1016/j.biopsycho.2008.06.004 18639608 PMC2596989

[B7] Brummett B. H. Krystal A. D. Siegler I. C. Kuhn C. Surwit R. S. Zuchner S.. (2007). Associations of a regulatory polymorphism of monoamine oxidase-A gene promoter (MAOA-uVNTR) with symptoms of depression and sleep quality. Psychosom. Med. 69, 396–401. 10.1097/psy.0b013e31806d040b 17585061 PMC2777888

[B8] Buckholtz J. W. Meyer-Lindenberg A. (2008). MAOA and the neurogenetic architecture of human aggression. Trends Neurosci. 31, 120–129. 10.1016/j.tins.2007.12.006 18258310

[B9] Byrd A. L. Manuck S. B. (2014). MAOA, childhood maltreatment, and antisocial behavior: meta-analysis of a gene-environment interaction. Biol. Psychiatry 75, 9–17. 10.1016/j.biopsych.2013.05.004 23786983 PMC3858396

[B10] Caspi A. McClay J. Moffitt T. E. Mill J. Martin J. Craig I. W.. (2002). Role of genotype in the cycle of violence in maltreated children. Science 297, 851–854. 10.1126/science.1072290 12161658

[B11] Cerasa A. Gioia M. C. Fera F. Passamonti L. Liguori M. Lanza P.. (2008). Ventro-lateral prefrontal activity during working memory is modulated by MAO A genetic variation. Brain Res. 1201, 114–121. 10.1016/j.brainres.2008.01.048 18294618

[B12] Chen K. Ou X. Chen G. Choi S. H. Shih J. C. (2005). R1, a novel repressor of the human monoamine oxidase A. J. Biol. Chem. 280, 11552–11559. 10.1074/jbc.m410033200 15654081 PMC2861901

[B13] Cullinan W. E. Ziegler D. R. Herman J. P. (2008). Functional role of local GABAergic influences on the HPA axis. Brain Struct. Funct. 213, 63–72. 10.1007/s00429-008-0192-2 18696110

[B14] Dedovic K. Duchesne A. Andrews J. Engert V. Pruessner J. C. (2009). The brain and the stress axis: the neural correlates of cortisol regulation in response to stress. NeuroImage 47, 864–871. 10.1016/j.neuroimage.2009.05.074 19500680

[B15] Dedovic K. Renwick R. Mahani N. K. Engert V. Lupien S. J. Pruessner J. C. (2005). The montreal imaging stress task: using functional imaging to investigate the effects of perceiving and processing psychosocial stress in the human brain. J. Psychiatry Neurosci. 30, 319–325. 16151536 PMC1197276

[B16] Denson T. F. Dobsonstone C. Ronay R. Von H. W. Schira M. M. (2014). A functional polymorphism of the MAOA gene is associated with neural responses to induced anger control. J. Cogn. Neurosci. 26, 1418–1427. 10.1162/jocn_a_00592 24564461

[B17] Doornbos B. Dijck-Brouwer D. A. J. Kema I. P. Tanke M. A. C. van Goor S. A. Muskiet F. A. J.. (2009). The development of peripartum depressive symptoms is associated with gene polymorphisms of MAOA, 5-HTT and COMT. Prog. Neuropsychopharmacol. Biol. Psychiatry 33, 1250–1254. 10.1016/j.pnpbp.2009.07.013 19625011

[B18] Fan M. Liu B. Jiang T. Jiang X. Zhao H. Zhang J. (2010). Meta-analysis of the association between the monoamine oxidase-A gene and mood disorders. Psychiatr. Genet. 20, 1–7. 10.1097/ypg.0b013e3283351112 20010318

[B19] Filipenko M. L. Beilina A. G. Alekseyenko O. V. Dolgov V. V. Kudryavtseva N. N. (2002). Repeated experience of social defeats increases serotonin transporter and monoamine oxidase A mRNA levels in raphe nuclei of male mice. Neurosci. Lett. 321, 25–28. 10.1016/s0304-3940(01)02495-8 11872248

[B20] Green J. G. McLaughlin K. A. Berglund P. A. Gruber M. J. Sampson N. A. Zaslavsky A. M.. (2010). Childhood adversities and adult psychiatric disorders in the national comorbidity survey replication I: associations with first onset of DSM-IV disorders. Arch. Gen. Psychiatry 67, 113–123. 10.1001/archgenpsychiatry.2009.186 20124111 PMC2822662

[B21] Grunewald M. Johnson S. Lu D. Wang Z. Lomberk G. Albert P. R.. (2012). Mechanistic role for a novel glucocorticoid-KLF11 (TIEG2) protein pathway in stress-induced monoamine oxidase A expression. J. Biol. Chem. 287, 24195–24206. 10.1074/jbc.m112.373936 22628545 PMC3397846

[B22] Higuchi Y. Soga T. Parhar I. S. (2017). Regulatory pathways of monoamine oxidase a during social stress. Front. Neurosci. 11:604. 10.3389/fnins.2017.00604 29163009 PMC5671571

[B23] Higuchi Y. Soga T. Parhar I. S. (2019). Social defeat stress decreases mRNA for monoamine oxidase A and increases 5-HT turnover in the brain of male nile tilapia (*Oreochromis niloticus*). Front. Pharmacol. 9:1549. 10.3389/fphar.2018.01549 30687104 PMC6333864

[B24] Holz N. Boecker R. Buchmann A. F. Blomeyer D. Baumeister S. Hohmann S.. (2016). Evidence for a sex-dependent MAOA× childhood stress interaction in the neural circuitry of aggression. Cereb. Cortex 26, 904–914. 10.1093/cercor/bhu249 25331606

[B25] Jabbi M. Korf J. Kema I. P. Hartman C. van der Pompe G. Minderaa R. B.. (2007). Convergent genetic modulation of the endocrine stress response involves polymorphic variations of 5-HTT, COMT and MAOA. Mol. Psychiatry 12, 483–490. 10.1038/sj.mp.4001975 17453062

[B26] Jung C. Greco S. Nguyen H. H. Ho J. T. Lewis J. G. Torpy D. J.. (2014). Plasma, salivary and urinary cortisol levels following physiological and stress doses of hydrocortisone in normal volunteers. BMC Endocr. Disord. 14:91. 10.1186/1472-6823-14-91 25425285 PMC4280712

[B27] Kageyama K. Tozawa F. Horiba N. Watanobe H. Suda T. (1998). Serotonin stimulates corticotropin-releasing factor gene expression in the hypothalamic paraventricular nucleus of conscious rats. Neurosci. Lett. 243, 17–20. 10.1016/s0304-3940(98)00097-4 9535102

[B28] Kalbitzer J. Kalbitzer U. Knudsen G. M. Cumming P. Heinz A. (2013). How the cerebral serotonin homeostasis predicts environmental changes: a model to explain seasonal changes of brain 5-HTT as intermediate phenotype of the 5-HTTLPR. Psychopharmacology 230, 333–343. 10.1007/s00213-013-3308-1 24150247

[B29] Kirschbaum C. Hellhammer D. H. (1989). Salivary cortisol in psychobiological research: an overview. Neuropsychobiology 22, 150–169. 10.1159/000118611 2485862

[B30] Kogler L. Gur R. C. Derntl B. (2015). Sex differences in cognitive regulation of psychosocial achievement stress: brain and behavior. Hum. Brain Mapp. 36, 1028–1042. 10.1002/hbm.22683 25376429 PMC6869735

[B31] Leonard B. E. (2005). The HPA and immune axes in stress: the involvement of the serotonergic system. Eur. Psychiatry 20, S302–S306. 10.1016/s0924-9338(05)80180-4 16459240

[B32] Li C. Sun X. Dong D. Zhong X. Wang X. Yao S. (2019). Effect of corticotropin-releasing hormone receptor1 gene variation on psychosocial stress reaction *via* the dorsal anterior cingulate cortex in healthy adults. Brain Res. 1707, 1–7. 10.1016/j.brainres.2018.11.020 30447186

[B33] Lucassen P. J. Pruessner J. Sousa N. Almeida O. F. Van Dam A. M. Rajkowska G.. (2014). Neuropathology of stress. Acta Neuropathol. 127, 109–135. 10.1007/s00401-013-1223-5 24318124 PMC3889685

[B34] Luo S. Yu D. Han S. (2016). Genetic and neural correlates of romantic relationship satisfaction. Soc. Cogn. Affect. Neurosci. 11, 337–348. 10.1093/scan/nsv117 26385612 PMC4733345

[B35] Ma R. Gan G. Zhang J. Ming Q. Jiang Y. Gao Y.. (2018). MAOA genotype modulates default mode network deactivation during inhibitory control. Biol. Psychol. 138, 27–34. 10.1016/j.biopsycho.2018.08.006 30092258

[B36] Marmorstein N. R. Hart D. (2011). Interactions between MAOA genotype and receipt of public assistance: predicting change in depressive symptoms and body mass index. J. Res. Adolesc. 21, 619–630. 10.1111/j.1532-7795.2010.00694.x 21949471 PMC3178327

[B37] McEwen B. S. Nasca C. Gray J. D. (2016). Stress effects on neuronal structure: hippocampus, amygdala, and prefrontal cortex. Neuropsychopharmacology 41, 3–23. 10.1038/npp.2015.171 26076834 PMC4677120

[B38] Meyer-Lindenberg A. Buckholtz J. W. Kolachana B. R. Hariri A. Pezawas L. Blasi G.. (2006). Neural mechanisms of genetic risk for impulsivity and violence in humans. Proc. Natl. Acad. Sci. U S A 103, 6269–6274. 10.1073/pnas.0511311103 16569698 PMC1458867

[B39] Ming Q. Zhong X. Zhang X. Pu W. Dong D. Jiang Y.. (2017). State-independent and dependent neural responses to psychosocial stress in current and remitted depression. Am. J. Psychiatry 174, 971–979. 10.1176/appi.ajp.2017.16080974 28618857

[B40] Morais M. Santos P. A. Mateus-Pinheiro A. Patricio P. Pinto L. Sousa N.. (2014). The effects of chronic stress on hippocampal adult neurogenesis and dendritic plasticity are reversed by selective MAO-A inhibition. J. Psychopharmacol. 28, 1178–1183. 10.1177/0269881114553646 25315831

[B41] Naoi M. Maruyama W. Shamoto-Nagai M. (2018). Type A monoamine oxidase and serotonin are coordinately involved in depressive disorders: from neurotransmitter imbalance to impaired neurogenesis. J. Neural Transm. 125, 53–66. 10.1007/s00702-017-1709-8 28293733

[B42] Ou X. M. Chen K. Shih J. C. (2006). Glucocorticoid and androgen activation of monoamine oxidase A is regulated differently by R1 and Sp1. J. Biol. Chem. 281, 21512–21525. 10.1074/jbc.m600250200 16728402

[B43] Passamonti L. Fera F. Magariello A. Cerasa A. Gioia M. C. Muglia M.. (2006). Monoamine oxidase-A genetic variations influence brain activity associated with inhibitory control: new insight into the neural correlates of impulsivity. Biol. Psychiatry 59, 334–340. 10.1016/j.biopsych.2005.07.027 16202396

[B44] Priess-Groben H. A. Hyde J. S. (2013). 5-HTTLPR X stress in adolescent depression: moderation by MAOA and gender. J. Abnorm. Child Psychol. 41, 281–294. 10.1007/s10802-012-9672-1 22836288

[B45] Pruessner J. C. Dedovic K. Khalili-Mahani N. Engert V. Pruessner M. Buss C.. (2008). Deactivation of the limbic system during acute psychosocial stress: evidence from positron emission tomography and functional magnetic resonance imaging studies. Biol. Psychiatry 63, 234–240. 10.1016/j.biopsych.2007.04.041 17686466

[B46] Sabol S. Z. Hu S. Hamer D. (1998). A functional polymorphism in the monoamine oxidase A gene promoter. Hum. Genet. 103, 273–279. 10.1007/s004390050816 9799080

[B47] Sacher J. Houle S. Parkes J. Rusjan P. Sagrati S. Wilson A. A.. (2011). Monoamine oxidase A inhibitor occupancy during treatment of major depressive episodes with moclobemide or St. John’s wort: an [^11^C]-harmine PET study. J. Psychiatry Neurosci. 36, 375–382. 10.1503/jpn.100117 21463543 PMC3201991

[B48] Semont A. Fache M. P. Ouafik L. Hery M. Faudon M. Hery F. (1999). Effect of serotonin inhibition on glucocorticoid and mineralocorticoid expression in various brain structures. Neuroendocrinology 69, 121–128. 10.1159/000054410 9986925

[B49] Soliman A. Udemgba C. Fan I. Xu X. Miler L. Rusjan P.. (2012). Convergent effects of acute stress and glucocorticoid exposure upon MAO-A in humans. J. Neurosci. 32, 17120–17127. 10.1523/jneurosci.2091-12.2012 23197705 PMC6621843

[B50] Stefanovic B. Spasojevic N. Jovanovic P. Jasnic N. Djordjevic J. Dronjak S. (2016). Melatonin mediated antidepressant-like effect in the hippocampus of chronic stress-induced depression rats: regulating vesicular monoamine transporter 2 and monoamine oxidase A levels. Eur. Neuropsychopharmacol. 26, 1629–1637. 10.1016/j.euroneuro.2016.07.005 27499503

[B51] Stroud C. B. Davila J. Moyer A. (2008). The relationship between stress and depression in first onsets versus recurrences: a meta-analytic review. J. Abnorm. Psychol. 117, 206–213. 10.1037/0021-843x.117.1.206 18266498

[B52] Sun X. Ma R. Jiang Y. Gao Y. Ming Q. Wu Q.. (2018). MAOA genotype influences neural response during an inhibitory task in adolescents with conduct disorder. Eur. Child Adolesc. Psychiatry 27, 1159–1169. 10.1007/s00787-018-1170-8 29855796

[B300] Sun X. Li C. Zhong X. Dong D. Ming Q. Gao Y. (2020). Influence of psychosocial stress on activation in human brain regions: moderation by the 5-HTTLPR genetic locus. Physiol Behav. 220:112876 10.10.1016/j.physbeh.2020.112876 32194071

[B53] Suri D. Teixeira C. M. Cagliostro M. K. C. Mahadevia D. Ansorge M. S. (2015). Monoamine-sensitive developmental periods impacting adult emotional and cognitive behaviors. Neuropsychopharmacology 40, 88–112. 10.1038/npp.2014.231 25178408 PMC4262911

[B54] Takao K. Nagatani T. Kitamura Y. Yamawaki S. (1997). Effects of corticosterone on 5-HT1A and 5-HT2 receptor binding and on the receptor-mediated behavioral responses of rats. Eur. J. Pharmacol. 333, 123–128. 10.1016/s0014-2999(97)01126-6 9314024

[B55] Ulrich-Lai Y. M. Herman J. P. (2009). Neural regulation of endocrine and autonomic stress responses. Nat. Rev. Neurosci. 10, 397–409. 10.1038/nrn2647 19469025 PMC4240627

[B56] Voltas N. Aparicio E. Arija V. Canals J. (2015). Association study of monoamine oxidase-A gene promoter polymorphism (MAOA-uVNTR) with self-reported anxiety and other psychopathological symptoms in a community sample of early adolescents. J. Anxiety Disord. 31, 65–72. 10.1016/j.janxdis.2015.02.004 25747527

[B57] Vors O. Marqueste T. Mascret N. (2018). The trier social stress test and the trier social stress test for groups: qualitative investigations. PLoS One 13:e195722. 10.1371/journal.pone.0195722 29641572 PMC5895062

[B58] Wang Z. Chen L. Rong X. Wang X. (2017a). Upregulation of MAOA in the hippocampus results in delayed depressive-like behaviors in burn mice. Burns [Epub ahead of print]. 10.1016/j.burns.2017.03.013 28413107

[B59] Wang Z. Chen L. Zhang L. Wang X. (2017b). Paradoxical sleep deprivation modulates depressive-like behaviors by regulating the MAOA levels in the amygdala and hippocampus. Brain Res. 1664, 17–24. 10.1016/j.brainres.2017.03.022 28365314

[B60] Winberg S. Lepage O. (1998). Elevation of brain 5-HT activity, POMC expression, and plasma cortisol in socially subordinate rainbow trout. Am. J. Physiol. 274, R645–R654. 10.1152/ajpregu.1998.274.3.r645 9530229

[B61] Zhang Y. Ming Q. Wang X. Yao S. (2016). The interactive effect of the MAOA-VNTR genotype and childhood abuse on aggressive behaviors in Chinese male adolescents. Psychiatr. Genet. 26, 117–123. 10.1097/ypg.0000000000000125 26945458

