# Supplementary material for: The MAOA Gene Influences the Neural Response to Psychosocial Stress in the Human Brain
Source: Front Behav Neurosci. 2020 May 15;14:65. doi: 10.3389/fnbeh.2020.00065 (PMC7243356; doi:10.3389/fnbeh.2020.00065)
Supplement: Supplementary file 1 [file Data_Sheet_1.docx]

**Supplementary material**

**The Montreal Imaging Stress Task**

The use of the Montreal Imaging Stress Task (MIST) is well established in stress research; it induces psychosocial stress using elements of uncontrollability and social evaluative threat (Dedovic et al., 2005). In this study, we administered the MIST in a block design with three imaging runs. Each run lasted 7 min and consisted of six blocks with three conditions. During the rest condition (30 s), used to record the baseline state, the program interface was displayed on the screen but the subject had tasks. He was asked to keep his eyes open and not to press a button until the next mental arithmetic task appeared. During the control condition (90 s), used to record brain activation related to the mental arithmetic aspects of the task in the absence of stress, the arithmetic problems were presented and subjects were asked to try to solve them as quickly and accurately as possible, with no time restriction. Average performance under the control condition could reach ~90%. During the experimental condition (90 s), problems were presented as in the control condition, and time pressure was induced by display of a time bar that adapted to each subject’s performance to enforce an accuracy rate of ~50%. Subjects received “correct” or “incorrect” feedback after providing the answer to each math question, and a simulative performance bar at the top of the screen showed that their accuracy rates were below that of the “average subject,” which was set artificially to 80%. When the subject provided no response within the time limit, the response “timeout” was displayed. The order of presentation in each run was rest—control—experimental—rest—control—experimental, with no interval between blocks.

Investigators provided scripted negative verbal feedback (~30 s) after each run segment via headphones, emphasizing the need for better performance to enhance the subjects’ perceived stress (Ming et al., 2017). They informed subjects about their performance and told them that their accuracy rates must be close to average, at minimum, if their data were to be used in the study. The subjects were also told that all investigators in the scanner room were following the their performance on a second monitor (Dedovic et al., 2005).

Participants’ subjective stress levels were assessed before and after the MIST using an orally administered 0–10 visual analog scale (0 = absence of stress, 10 = maximal stress), and subjective stress changes were calculated by subtracting pre-MIST from post-MIST scores (Ming et al., 2017).

**Cortisol measurement**

Saliva samples were collected with a Salivette (Sarstedt, Nümbrecht, Germany) at participants’ time of arrival (Cort1), after 30 min of rest (Cort2), just before entering the scanner (Cort3, about 10 min after Cort2), during anatomical imaging (Cort4), after each of the three MIST runs (Cort5–7), and immediately upon leaving the scanner (Cort8, ~25 min after MIST completion). Subjects were given instructions for the task for the first 7–8 min of the 30-min rest period (Cort2), then asked to sit and rest, with no vigorous exercise allowed. To control for circadian fluctuations, scanning was performed between 2:00 and 5:00 pm (Ming et al., 2017). Cortisol concentrations were quantitated with a human cortisol enzyme-linked immunosorbent assay kit (Bio-Swamp, Shanghai, China). To obtain a summary measure of cortisol responses independent of the precision of measurement times, we subtracted Cort4 (pre-MIST baseline) from Cort8 (expected highest cortisol level), as described previously (Ming et al., 2017).

**References**

Dedovic, K., Renwick, R., Mahani, N. K., Engert, V., Lupien, S. J., Pruessner, J. C., 2005. The Montreal Imaging Stress Task: using functional imaging to investigate the effects of perceiving and processing psychosocial stress in the human brain. Journal of Psychiatry and Neuroscience 30, 319-325.

Ming, Q., Zhong, X., Zhang, X., Pu, W., Dong, D., Jiang, Y., Gao, Y., Wang, X., Detre, J. A., Yao, S., Rao, H., 2017. State-Independent and Dependent Neural Responses to Psychosocial Stress in Current and Remitted Depression. AMERICAN JOURNAL OF PSYCHIATRY 174, 971-979.

**Supplementary Table 1. Brain regions showing activation/deactivation under the experimental condition compared with the control condition**

| **Brain region** | **Side** | **MNI coordinates** | | | **Cluster size** | ***t*** | ***P* _FWE corrected_** |
| --- | --- | --- | --- | --- | --- | --- | --- |
|  |  | **x** | **y** | **z** |  |  |  |
| ***Activation*** | | | | | | | |
| Temporal lobe | L/R | -15 | -3 | 63 | 20158 | 16.56 | <0.05 |
| Occipital lobe, |  | | | | | | |
| Frontal lobe, |  |  |  |  |  |  |  |
| cingulate |  |  |  |  |  |  |  |
| Insula |  |  |  |  |  |  |  |
| Thalamus |  |  |  |  |  |  |  |
| Insula | L | -30 | 21 | 3 | 851 | 12.62 | <0.05 |
| Middle Frontal Gyrus  Inferior Frontal Gyrus |  |  |  |  |  |  |  |
|  |  |  |  |  |  |  |  |
| ***Deactivation*** |  |  |  |  |  |  |  |
| Temporal Lobe/ Hippocampus | R | 42 | 9 | -36 | 156 | -12.53 | <0.05 |
| Temporal Lobe/ Hippocampus | L | -45 | 9 | -30 | 56 | -8.61 | <0.05 |
| vmPFC/ subgenual ACC | L/R | 12 | 27 | -9 | 119 | -10.77 | <0.05 |
| Angular | L | -48 | -75 | 39 | 92 | -9.14 | <0.05 |
| Angular | R | 48 | -72 | 45 | 82 | -9.56 | <0.05 |

Significance was determined based on voxel-wise, familywise error–corrected *p* < 0.05.

Abbreviation: ACC,anterior cingulate cortex; vmPFC, ventromedial prefrontal cortex.

**Supplementary Table 2. Brain regions showing decreased functional connectivity with the anterior hippocampus under the experimental condition compared with the control condition**

| **Brain region** | **Side** | **MNI coordinates** | | | **Cluster size** | ***t*** | ***P* _FWE corrected_** |
| --- | --- | --- | --- | --- | --- | --- | --- |
|  |  | **x** | **y** | **z** |  |  |  |
| ***Left anterior hippocampus*** | | | | | | | |
| Precuneus/ Posterior Cingulate | L/R | -36 | -93 | -6 | 2037 | 6.98 | <0.05 |
| Parahippocampa Gyrus/ Lingual Gyrus | L | -12 | -39 | -6 | 133 | 6.59 | <0.05 |
| vmPFC /Anterior Cingulate | L/R | -3 | 48 | 0 | 652 | 6.80 | <0.05 |
| Lingual Gyrus | L | -6 | -78 | -12 | 37 | 5.77 | <0.05 |
| MTG/STG | R | 48 | -57 | -12 | 375 | 6.45 | <0.05 |
| MTG | L | -60 | -15 | -18 | 41 | 6.20 | <0.05 |
| MTG/ITG | R | 54 | -3 | 21 | 90 | 5.72 | <0.05 |
| ***Right anterior hippocampus*** | | | | | | | |
| Posterior Cingulate/ Precuneus | R | 0 | -48 | 27 | 2756 | 7.79 | <0.05 |
| STG/MTG/IPL | R | 66 | -15 | 0 | 1230 | 7.68 | <0.05 |
| Parahippocampa Gyrus | R | 24 | -33 | -27 | 38 | 6.09 | <0.05 |
| vmPFC /Anterior Cingulate | L/R | 6 | 54 | 0 | 651 | 7.17 | <0.05 |
| Lingual Gyrus | R | 27 | -66 | -6 | 91 | 5.88 | <0.05 |
| Parahippocampa Gyrus/ IFG | L | -27 | 6 | -21 | 120 | 6.67 | <0.05 |
| MTG | L | -51 | -6 | -21 | 219 | 6.69 | <0.05 |

Significance was determined based on voxel-wise, familywise error–corrected *p* < 0.05.

Abbreviation: IFG, inferior frontal gyrus; IPL, inferior parietal lobule; ITG, inferior temporal gyrus; MTG, middle temporal gyrus; STG, superior temporal gyrus; vmPFC, ventromedial prefrontal cortex.

**Supplementary Table 3. Brain regions showing decreased functional connectivity with the anterior hippocampus under the experimental condition compared with the control condition, in an analysis with the cortisol level and anxiety score serving as covariates**

| **Brain region** | **Side** | **MNI coordinates** | | | **Cluster size** | ***t*** | ***P* _FWE corrected_** |
| --- | --- | --- | --- | --- | --- | --- | --- |
|  |  | **x** | **y** | **z** |  |  |  |
| ***Left anterior hippocampus*** | | | | | | | |
| Precuneus/ Posterior Cingulate | L/R | -36 | -39 | -6 | 1974 | 7.12 | <0.05 |
| Parahippocampa Gyrus/ Lingual Gyrus | L | -21 | -33 | -15 | 145 | 6.93 | <0.05 |
| vmPFC /Anterior Cingulate | L/R | -3 | 48 | 0 | 694 | 6.69 | <0.05 |
| Lingual Gyrus | L | -6 | -78 | -12 | 30 | 5.54 | <0.05 |
| MTG/STG | R | 48 | -57 | -15 | 378 | 6.67 | <0.05 |
| MTG | L | -60 | -15 | -18 | 34 | 5.96 | <0.05 |
| MTG/ITG | R | 54 | -3 | 21 | 84 | 5.57 | <0.05 |
| ***Right anterior hippocampus*** | | | | | | | |
| Posterior Cingulate/ Precuneus | R | 3 | -51 | 27 | 2709 | 7.46 | <0.05 |
| STG/MTG/IPL | R | 66 | -15 | 0 | 1199 | 7.73 | <0.05 |
| Parahippocampa Gyrus | R | 24 | -33 | -27 | 57 | 6.01 | <0.05 |
| vmPFC /Anterior Cingulate | L/R | 6 | 54 | 0 | 580 | 6.86 | <0.05 |
| Lingual Gyrus | R | 27 | -75 | -15 | 91 | 5.86 | <0.05 |
| Parahippocampa Gyrus/ IFG | L | -27 | 6 | -21 | 110 | 6.44 | <0.05 |
| MTG | L | -51 | -6 | -21 | 214 | 6.40 | <0.05 |

Significance was determined based on voxel-wise, familywise error–corrected *p* < 0.05.

Abbreviation: IFG, inferior frontal gyrus; IPL, inferior parietal lobule; ITG, inferior temporal gyrus; MTG, middle temporal gyrus; STG, superior temporal gyrus; vmPFC, ventromedial prefrontal cortex.

**Supplementary Table 4. Characteristics of greater activation of the right anterior hippocampus in L allele carriers**

| **Brain region** | **Side** | **MNI coordinates** | | | **Cluster size** | ***t*** | ***P* _uncorrected_** | ***P* _FWE corrected_** |
| --- | --- | --- | --- | --- | --- | --- | --- | --- |
|  |  | **x** | **y** | **z** |  |  |  |  |
| Anterior hippocampus | R | 27 | -12 | -18 | 144 | 5.21 | <.001 | .028 |

Data are derived from group comparison in the first-level general linear model only including the control and experimental conditions.


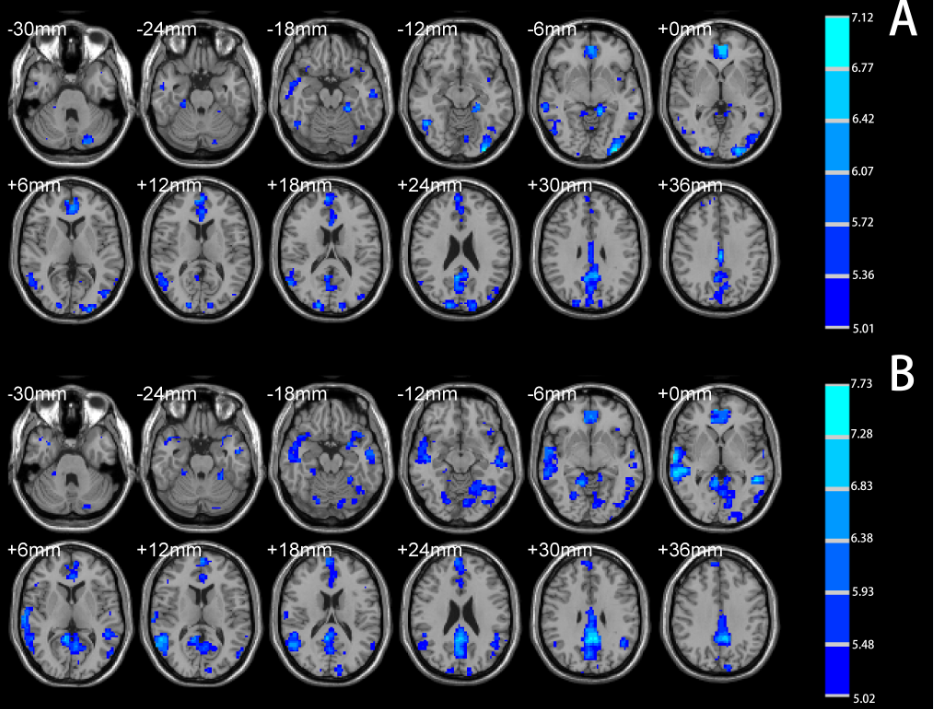


**Supplementary Figure 1** Brain regions showing significantly decreased functional connectivity with the left (A) and right (B) anterior hippocampus under the stress condition compared with the control condition in analyses with the cortisol level and anxiety score serving as covariates (total cohort). Significance was determined based on voxel-wise, familywise error–corrected p < 0.05; the colored bar indicates the t statistic.
